# Supplementary material for: Fitting a lattice model with local and global transmission to spread of a plant disease
Source: PLoS Comput Biol. 2026 Feb 18;22(2):e1013404. doi: 10.1371/journal.pcbi.1013404 (PMC12945315; doi:10.1371/journal.pcbi.1013404)
Supplement: Attachment — Submitted filename: Review_Best_and_Cunniffe.pdf [file pcbi.1013404.s007.pdf]

Review of "Fitting a lattice model with local and global transmission to spread of a plant disease"

Alex Best and Nik J. Cunniffe

November 2025

Summary

This article develops and applies a lattice-based model using a pair approximation method to the spatial spread of a plant disease, with a case study on Bahia bark scaling in citrus. The authors successfully fit this model to experimental field data, demonstrating that it captures the disease dynamics effectively and identifies a significant component of local, near-neighbour transmission. Its performance is shown to be comparable to a more established dispersal-kernel modelling approach. From my perspective, having substantially checked the literature, I can confirm that while pair approximation models have a strong theoretical foundation in epidemiology, I could not indeed find a preceding instance where such a model was formally fitted to real-world data for a specific pathosystem. This successful application constitutes the main novelty of the work and makes it of high importance to the literature. It represents a substantial addition to the methodological toolkit of mathematical ecology and epidemiology.

One direct and impactful application can be that it provides a pathway for embedding realistic spatial dynamics into decision support systems. Indeed, this lattice-based pair approximation models with local:global ratio consideration and its fitting offer a deterministic framework that can realistically capture the spatial disease spread dynamics often associated with more computationally intensive stochastic models. This bridge between realism and computational efficiency is a significant step forward for optimizing disease control strategies in plant epidemiology. Therefore, I strongly recommend this paper for publication, pending revisions. My concerns are largely minor, and some are merely suggestions that can be ignored if they require undue additional work. I will specify these when it applies.

Comments

1. (Minor) Line 100-101: "those trees initially inoculated at the start of the experiment, one row outside of the main tree stand" sounds like the founders are out of the trellis, and do not resonate well with the relationship $P_S+P_E+P_I+P_F = 1$. The little ambiguity should be cleared up. From my understanding, the full lattice is made of (a) a main tree stand and (b) a founder row.

2. (Minor) In line with the previous comment, although it is well described in lines 163-173, may be specifying that the dark rows stand for founders in Fig 4 and 7 captions would help "skimming readers".

3. (Typo) In Eq (1), $L_F q_{I/S} \to L q_{I/S}$, considering lines 112-114.

4. (Minor) Line 122: "since each individual has 4 neighbours" assumes a Von Neumann neighbourhood. To what extent would the results change if a Moore neighbourhood (8 neighbours) was used? This questions the fundamental distance considered for neighbouring, specifically whether a unit of distance is distinct from that same unit multiplied by $\sqrt{2}$

(Pythagoras). Have you tested both configurations to see how they impact the model approximation and calibration? If further exploration is not deserved, this limitation deserves at least a brief discussion in the methods.

5. (Typo) Eq (6) $\gamma \to \rho$.
6. (Major) The introduction of the local:global infection parameter control L looks quite simplistic as it is curious that it is a simple ratio. Can it be more elaborated upon its introduction? More explicitely, the distance structure is ignored and all "global" infections seem to have the same probability; all non-local infections are lumped together, ignoring spatial decay. I suggest the single L to be introduced in the singlet model as a two-bin approximation neighbours vs "everyone-else", which is acknowledgely useful for minimal models but might miss clustering effects and realistic spatial spread. Then maybe it can become a better approximation through the pair closure and/or the data fitting.
7. (Minor) Lines 189-191: I do not understand "For these purposes (...) tree stand".
8. (Minor) Line 199: The link is truncated.
9. (Minor) Line 203: Which threshold? The sentence "Table 1 shows how the number of successful fits depends on this threshold" cannot stand alone. Additionally, although the threshold was introduced at lines 191-192, it needs more details; otherwise, it is difficult to understand.
10. (Typo) Fig. 1 (c) subcaption: $\to$ Latent period, $1/\rho$
11. (Typo) Fig. 2: Looks like the author's comment (Nik) is still visible in the caption. (Minor): If the authors deem it useful, I would suggest adding a legend to Fig. 2 (a) as well as a posterior predictive mean (PPM) line. A mean curve is even more relevant for Fig. 3 (a) and Fig. 6 left, because of the variation. Fig. 2 caption can also be made richer for "skimming readers".
12. (Major) Fig 4.: The choice of L, β, β_F, ρ, δ out of their 95% confidence interval (Table 2) for the simulation is curious. What justifies this choice?
13. (Minor) Lines 235-236: The notion of threshold here is still unclear, as previously.
14. (Minor) Line 239: What does "only 5 parameter sets accepted where $\alpha > 5$" mean?
15. (Major) Lines 245-248: We need a better explanation of why the PPC of the dispersal model look visually more similar to the stochastic simulation of the lattice model than the actual dispersal model.

    Additionally, I suggest a single, robust measure to compare the ability of the lattice and dispersal models to fit the data: the Earth Mover's Distance (EMD) applied to monthly snapshots. The EMD quantifies the minimum cost to transform one spatial distribution into another, providing a powerful metric for overall spatial dissimilarity[Rubner et al. 2000: The earth mover's distance as a metric for image retrieval. IJCV 40(2)]. Since it accounts for the full spatial structure, it offers a more comprehensive comparison than metrics based solely on global or row-wise infection proportions (Figs. 3 and 6). Hence, plotting $EMD(lattice, data)(t)$ and $EMD(dispersal, data)(t)$, t in months, on the same graph would allow for a direct visual and quantitative discussion of their relative performance over time.

    While this is not a mandatory request, I believe its inclusion would significantly strengthen the manuscript's results and provide a concrete basis for discussing the advantages of the lattice model. The EMD could, of course, be substituted with a similar spatial metric, such as a class-wise Hausdorff distance or normalized Hamming distance [Hamming 1950: Error detecting and error correcting codes. The Bell System Technical Journal 29(2)]. Given the nature of the experimental data, latently infected trees can be considered in either the S or I class while assessing the EMD, Hausdorff, Hamming, or a similar metric. This necessary misclassification could influence the results and may be worth a brief mention.
