# Supplementary material for: Fitting a lattice model with local and global transmission to spread of a plant disease
Source: PLoS Comput Biol. 2026 Feb 18;22(2):e1013404. doi: 10.1371/journal.pcbi.1013404 (PMC12945315; doi:10.1371/journal.pcbi.1013404)
Supplement: Attachment — Submitted filename: ReviewerComments.docx [file pcbi.1013404.s006.docx]

This work demonstrates the applicability of a lattice-based mathematical approach to model the spread of Bahia bark scaling of citrus (BBSC). Spatially explicit approaches such as these are routinely employed to develop theoretical ideas in the field. This epidemiological compartmental approach is validated against an established dispersal-kernel model, and both models are fit to experimental data for BBSC using a well-supported Bayesian paradigm. Predictions generated from this model are used to demonstrate effectiveness of the approach for determining the number of infested locales in the studied domain of interest. These results are further supported by direct comparison to predictions from the dispersal-kernel model. The authors conclude with a discussion of the broader applications of this technique to many economically and ecologically significant plant pests and pathogens. We read the paper with great interest. Below we provide some constructive comments that we feel could improve the manuscript before publication:

Abstract:

- No major comments, a representative summary of the manuscript contents.

Introduction:

- Overall, very well motivated and explained. I personally would like to have more details on the BBSC pathogen – the reader having a better understanding of the disease might help motivate some of the later modelling choices.
- Perhaps more details could be given about the advantages of the lattice-based model around lines 76-83 and what kind of pathogens they would be appropriate for. (It is touched upon that they are appropriate for crops in production settings, but I’m not left with a strong feeling about why this type of model in this setting).
- More detail could be provided on the results of [59] which seems to underpin the individual-based model with the dispersal kernel (what was done in [59] and what is new in this manuscript with the dispersal kernel model?)

Materials and Methods

- Leading with an explanation of the experimental data might help structure this section. The model could then be introduced referring to the data to defend certain model choices. In general, I found myself asking questions that were then answered one-two paragraphs later (e.g., why one row of founder trees? What does the experimental data look like?) so maybe a slight restructure could help with the clarity.
- Maybe it should be emphasised that the model is not for the pathogen itself but for the underlying disease compartments.
- [101] Not sure what is meant by a “main tree stand”
- How sensitive are the results to the choice of initial conditions? The founder trees do have different parameter choices for the rate of infection, but how is this decided and what impact would it have on the results?
- More explanation could be given to how equations (1) and (2) are derived.
- I’d suggest having a separate section with a new subheading for each model description.
- I would like to have seen some Figures to aid understanding of each model. My suggestion would be to show a Figure for the experimental data (currently S1 in the Supplementary Information but I see no reason this shouldn’t be in the main text), and a Figure to illustrate both models.
- In Figure S1, why is month 30 the first starting point to see some disease – I’m unclear about when the observations happened. 79 months is six and a half years, but the text says the observations were taken over a period of five years. This timeline is mirrored in the simulations (start at month 30).
- The model fitting/inference section is well explained in a succinct manner. Did you consider using any other fitting functions instead of sum of squared error?
- I think the introduction of the ‘threshold’ could be treated more carefully. At first it was unclear to me what this was, and that it was set by the authors – how is the threshold chosen and why? How are the “errors” on P_I and P_II calculated exactly and how does this lead to the threshold choice?

Results

- Results section could start with some succinct introduction to refresh readers (or for those jumping straight in at Results) – begins by talking about “this threshold” and I’d already forgotten what that was and had to go back!
- Tables 1 and 3 set out the acceptance rates for each model for different error thresholds. If I understand correctly, the error threshold is set by the authors, and it makes sense that a stricter (smaller) threshold results in a lower acceptance rate within the inference scheme. However, the final acceptance rates for the thresholds chosen are tiny – can the authors explain more about why they chose these thresholds? This will have a big impact on the posterior distributions – for example in Figure 1 the number of samples is actually very low (187). I’m not sure I agree with the authors that they have “a high enough number of acceptances to form smooth distributions”.
- [195] alpha in the dispersal model is taken to be between [0,10] – why this choice? (In addition, we had some confusion over the unit scales between the model and experiment, could perhaps be refined in the explanation here).
- [205] “bootstrap confidence intervals” Given the adopted Bayesian approach, should these be credible intervals instead?
- [207] “For the transmission parameters” perhaps a reminder of which parameters these are to help reader who may have jumped in at Results.
- [213-221] Many great points made re interpretation of results but would perhaps be better placed in the Discussion in favour of a condensed Results section.
- [216] Not sure if the Results section is an appropriate place to introduce the use of posterior predictive checks. May be helpful to include a short summary (at end of Methods) to indicate how the models are validated and compared.
- Have you plotted/considered corner plots for examining the posterior parameters?
- Comparison of run times feels like not a substantial result – would it be better commented upon in the discussion instead? Have the authors considered GPU acceleration for the dispersal model?

Discussion:

- The discussion is a good summary of the key findings, and we agree with the authors suggestions for future application of these approaches.
- [275] Sometimes referring to the non-lattice model as a dispersal kernel model or spatially explicit model. May be helpful to give these models consistent names.

Minor comments:

- Apostrophe typesetting issues throughout with tex ‘ instead of `
- [53] “Worryingly, a very large number… Risk Register” reads as though the concern is the fact we’ve documented them. Suggested “Documented and ranked in the UK Plant Health Risk Register are a worryingly large number of pathogens which could potentially invade and threaten UK forests in the future”.
- [71] no comma needed after “…individual based models [41,42,43]”
- [85] is “local:global” standard notation? Might be clearer to someone less familiar as “local-to-global”.
- [121] “its” should be “it’s” or “it is”.
- [126-127] add commas or semicolons to end of each equation.
- Figure 2 caption needs describing more explicitly (no mention of the points, the lines etc). What are panel (a) and (b)?
- Could the plot colours more intuitively represent the disease? (The black line could easily be missed as the founder trees and the red/blue colours don’t automatically read as susceptible and infected landscape to me).
- Some Figure captions are incomplete, Figure S1, Figure 4 and Figure 7 caption don’t describe the black line of founder trees.
- Figure 1 is in Materials and Methods before it’s mentioned – should be in the Results section.
- Figure 5 “Top row” in caption unneeded as figure only has one row.
- [181] “numbers infected” is unclear – maybe “number of infected”?
- [221 – 222] Figure 2 contains blue text and a superscript which looks like it may be a draft macro – remove?
- [232] “As a comparison” reads as though the examination itself is for comparison, rather than the use of the dispersal model generally.
- [237] Capitalise Table (and throughout for Fig/Table etc…)
- [288] “diseased” should be “diseases”?
- [300] oak processionary moth (no capitalisation)

We believe this work to be of high quality and of significant importance to the field, providing novel insights into the spread of BBSC and motivating additional developments and applications. The adopted methodology is flexible enough to be applied to other economically and ecologically significant plant pests and pathogens. We thank the authors in advance for the consideration of our comments and look forward to reading the next iteration of the manuscript.
