# Supplementary material for: Fitting a lattice model with local and global transmission to spread of a plant disease
Source: PLoS Comput Biol. 2026 Feb 18;22(2):e1013404. doi: 10.1371/journal.pcbi.1013404 (PMC12945315; doi:10.1371/journal.pcbi.1013404)
Supplement: Attachment — Submitted filename: ReviewerResponses.docx [file pcbi.1013404.s008.docx]

Dear editors and reviewers,

Many thanks for all of the helpful, constructive comments on our previous submission. We were pleased with all of the positive comments and found the constructive criticisms very useful. We are very pleased to now submit our revision for further consideration and believe it is much improved. Below we provide a point-by-point response to each of the three reviewers. We also start with a brief summary of our response to the two key themes of the reviews identified by the associate editor.

*Justifying choice of error threshold for the ABC fitting*

While there is no hard and fast rule for choosing a particular value for the acceptance threshold in ABC, we agree we had not given our particular choice enough justification or exploration. We have added further plots in the supplementary information of posterior distributions with different choices of threshold for comparison and we have also added to the text to explain our choice further.

*Presenting credible intervals vs confidence intervals for the parameters*

We thank the reviewers for pointing out our unusual choice of statistic to present. As suggested, we now provide credible intervals for the parameters using the 90% Smallest Credible Interval (or Highest Density Interval).

Linked to both points, we have also taken the opportunity to significantly increase the number of model runs used in our model fitting for both models by an order of 10, and also expand the ranges of a couple of the parameters, lending more statistical power to all of our results.

We look forward to hearing from you again regarding the manuscript.

Many thanks,

Alex Best & Nik Cunniffe

**Reviewer 1**

**This work demonstrates the applicability of a lattice-based mathematical approach to model the spread of Bahia bark scaling of citrus (BBSC). Spatially explicit approaches such as these are routinely employed to develop theoretical ideas in the field. This epidemiological compartmental approach is validated against an established dispersal-kernel model, and both models are fit to experimental data for BBSC using a well-supported Bayesian paradigm. Predictions generated from this model are used to demonstrate effectiveness of the approach for determining the number of infested locales in the studied domain of interest. These results are further supported by direct comparison to predictions from the dispersal-kernel model. The authors conclude with a discussion of the broader applications of this technique to many economically and ecologically significant plant pests and pathogens. We read the paper with great interest. Below we provide some constructive comments that we feel could improve the manuscript before publication:**

**Abstract:**

- **No major comments, a representative summary of the manuscript contents.**

Thanks.

**Introduction:**

- **Overall, very well motivated and explained. I personally would like to have more details on the BBSC pathogen – the reader having a better understanding of the disease might help motivate some of the later modelling choices.**

As mentioned below, we have now moved the methods subsection on the experimental data to the start of that section, and we think this will also help with the point here. Concerning the BBSC pathogen itself, as was already stated in the m/s (L95 of the revised m/s), the disease’s etiology is in fact unknown, i.e., the causal agent has not been identified. So, it is difficult to give more details on the pathogen - despite recent suggestions (Castro Gama et al., 2025, Crop Protection) the causal agent might be fungal, the evidence is currently inconclusive, and so we do not wish to speculate.

- **Perhaps more details could be given about the advantages of the lattice-based model around lines 76-83 and what kind of pathogens they would be appropriate for. (It is touched upon that they are appropriate for crops in production settings, but I’m not left with a strong feeling about why this type of model in this setting).**

We have edited some of this section to be clearer on their relevance for plant systems (P4 L77):

*“The assumptions of these models are highly appropriate to plant systems, especially managed and agricultural populations where plants are often laid out in regular spacings.”*

- **More detail could be provided on the results of [59] which seems to underpin the individual-based model with the dispersal kernel (what was done in [59] and what is new in this manuscript with the dispersal kernel model?)**

We have added some text here with a little more detail, and we note that we give much more information as we talk through the models in the methods section (P5 L98).

*“They showed their model could fit the BBSC data well, giving us a good comparison for our parameter estimates.”*

**Materials and Methods**

- **Leading with an explanation of the experimental data might help structure this section. The model could then be introduced referring to the data to defend certain model choices. In general, I found myself asking questions that were then answered one-two paragraphs later (e.g., why one row of founder trees? What does the experimental data look like?) so maybe a slight restructure could help with the clarity.**

We have switched this round as suggested (P5 L101).

- **Maybe it should be emphasised that the model is not for the pathogen itself but for the underlying disease compartments.**

We have added that this is a compartmental model (P6 L115)

- **[101] Not sure what is meant by a “main tree stand”**

We have changed this to “*main experimental tree stand*”, which is hopefully clearer (P6 L119).

- **How sensitive are the results to the choice of initial conditions? The founder trees do have different parameter choices for the rate of infection, but how is this decided and what impact would it have on the results?**

We have not investigated this directly. Most of the initial conditions are directly inferred from the experiment. Of course, the layout of the founder trees is much simplified in the model, but we believe this is well accounted for in having the separate transmission rate. As mentioned, we did look at also including a separate local parameter for the founder trees but it was reliably similar to the secondary local parameter so we felt justified in not including it.

- **More explanation could be given to how equations (1) and (2) are derived.**

These two equations are effectively just the standard S-E-I model, but with the addition of the local infection terms. We have added a note that when L=1 this is precisely the mean-field SEI model (P6 L133), which we hope along with the original explanations will make it clearer.

*“If L = 1 the equations (1)-(2) are simply the standard mean-field SEI model.”*

- **I’d suggest having a separate section with a new subheading for each model description.**

We thought about this but are wary of breaking up the methods section into too many subsections.

- **I would like to have seen some Figures to aid understanding of each model. My suggestion would be to show a Figure for the experimental data (currently S1 in the Supplementary Information but I see no reason this shouldn’t be in the main text), and a Figure to illustrate both models.**

To save space we feel it is best to keep the experimental figure in the SI (especially since this is more or less a reproduction of others’ work). We did try out producing some figures to illustrate the models, but felt that what we produced was no more helpful than the existing snapshots combined with the model descriptions.

- **In Figure S1, why is month 30 the first starting point to see some disease – I’m unclear about when the observations happened. 79 months is six and a half years, but the text says the observations were taken over a period of five years. This timeline is mirrored in the simulations (start at month 30).**

We apologise for not explaining this clearly. As we now state, the first infection was identified around 30 months after the experiment as started, and the five year span of regular inspections then started (P5 L109).

*“The first infection was detected around 30 months after the experimental grove was planted. “*

- **The model fitting/inference section is well explained in a succinct manner. Did you consider using any other fitting functions instead of sum of squared error?**

We did consider other measures when first starting the project, but went with sum of squares since it is a fairly standard approach to use. We suspect most choices of error would ultimately lead to similar posteriors.

- **I think the introduction of the ‘threshold’ could be treated more carefully. At first it was unclear to me what this was, and that it was set by the authors – how is the threshold chosen and why? How are the “errors” on P_I and P_II calculated exactly and how does this lead to the threshold choice?**

This was one of the two comments all the reviewers mentioned. We have added a brief bit of text in the introduction to add more detail (P9 L201), comment further on our choice of default value at the start of the results (P10 L217) and have added plots to the supplementary information where we show the posteriors for different choices of acceptance threshold.

*“Clearly, the posteriors will vary depending on the chosen acceptance threshold. We present results in the main text for a single chosen threshold, but show results for other values in the supplementary information and comment on our choice of default acceptance threshold.”*

*“In the supplementary information we show posterior distributions for a number of different thresholds for comparison. We find that our chosen threshold has enough accepted fits to form `smooth' posteriors, and the distributions remain consistent for small changes to the threshold.”*

**Results**

- **Results section could start with some succinct introduction to refresh readers (or for those jumping straight in at Results) – begins by talking about “this threshold” and I’d already forgotten what that was and had to go back!**

We have added an opening sentence to the results as suggested (P10 L215).

- **Tables 1 and 3 set out the acceptance rates for each model for different error thresholds. If I understand correctly, the error threshold is set by the authors, and it makes sense that a stricter (smaller) threshold results in a lower acceptance rate within the inference scheme. However, the final acceptance rates for the thresholds chosen are tiny – can the authors explain more about why they chose these thresholds? This will have a big impact on the posterior distributions – for example in Figure 1 the number of samples is actually very low (187). I’m not sure I agree with the authors that they have “a high enough number of acceptances to form smooth distributions”.**

As above, we have now added plots of the posteriors for other thresholds and some discussion of our choice of default value. We have also taken the opportunity to significantly increase the number of model runs (by an order of 10) and we also expanded the bounds of two of the parameters’ priors.

- **[195] alpha in the dispersal model is taken to be between [0,10] – why this choice? (In addition, we had some confusion over the unit scales between the model and experiment, could perhaps be refined in the explanation here).**

Obviously there is no *a priori* upperbound for alpha so we chose 10 assuming that it was unlikely to be any higher (as the posteriors show, a maximum value of 5-6 would have sufficed but we have kept the full posterior for transparency). We have added some detail to both the description of the experiment (P5 L103) and of the parameter alpha (P10 L207), which hopefully makes the unit scales clearer.

*“In the experiment underlying this work, a 15 × 16 grid of initially immature citrus trees was established with a 2m x 2m spacing adjacent to a small stand of older trees showing BBSC symptoms.”*

*“α has units of distance, with 2α the mean dispersal distance, but in the model one ‘unit’ of distance equates to 2 metres in the experiment.”*

- **[205] “bootstrap confidence intervals” Given the adopted Bayesian approach, should these be credible intervals instead?**

Yes, this was the other main point that all the reviewers mentioned. As discussed at the top of this response, we now present the 90% smallest credible intervals for the parameter values (P11 Table 1 and P15 Table 2).

- **[207] “For the transmission parameters” perhaps a reminder of which parameters these are to help reader who may have jumped in at Results.**

Changed as suggested (P11 L224).

*“For the two transmission parameters - βF for infections from founders and β for all secondary infections - the distributions are notably different…”*

- **[213-221] Many great points made re interpretation of results but would perhaps be better placed in the Discussion in favour of a condensed Results section.**

We considered this but our preference is to leave this discussion here as we think they are important context for understanding the results.

- **[216] Not sure if the Results section is an appropriate place to introduce the use of posterior predictive checks. May be helpful to include a short summary (at end of Methods) to indicate how the models are validated and compared.**

Given this is a fairly brief description – ultimately we just run 100 simulations using parameters from the posterior distributions – we felt it was best to leave this here.

- **Have you plotted/considered corner plots for examining the posterior parameters?**

We had included scatter plots of the correlations between the posteriors in the supplementary information, which are essentially a simplified version of corner plots. We feel this gives enough information about the relationships between parameters as it is, though we have added a little to the relevant sentences in the main text where these are mentioned (P11 L227).

*“We show correlations between the parameters in supplementary Fig. 2, which highlight that $\beta$ and $1/\rho$ have a strong non-linear correlation, with a much broader spread of transmission rates for high latent periods. We also see that low $L$ values require low $\beta$ values.”*

- **Comparison of run times feels like not a substantial result – would it be better commented upon in the discussion instead? Have the authors considered GPU acceleration for the dispersal model?**

We agree it is of less importance than the main results – which is why it was placed at the end – but it did seem worth reporting within the results section as it is potentially important for readers looking to reproduce our work or do something similar for their own data. We did not look at any more advanced regimes for running our code, as running a number of parallel simulations on an HPC was reasonable for our means. We have commented in the discussion about how this could be improved (P19 L322).

*“While more advanced computational techniques would improve the speed - utilising GPUs for example - this time difference between the approaches will always remain to some extent.”*

**Discussion:**

- **The discussion is a good summary of the key findings, and we agree with the authors suggestions for future application of these approaches.**

Thanks!

- **[275] Sometimes referring to the non-lattice model as a dispersal kernel model or spatially explicit model. May be helpful to give these models consistent names.**

We have re-phrased this sentence to try and keep the consistent label of the “dispersal model” (P18 L289).

**Minor comments:**

- **Apostrophe typesetting issues throughout with tex ‘ instead of `**

We have hopefully corrected all of these.

- **[53] “Worryingly, a very large number… Risk Register” reads as though the concern is the fact we’ve documented them. Suggested “Documented and ranked in the UK Plant Health Risk Register are a worryingly large number of pathogens which could potentially invade and threaten UK forests in the future”.**

Changed as suggested (P3 L53).

- **[71] no comma needed after “…individual based models [41,42,43]”**

Removed (P4 L71).

- **[85] is “local:global” standard notation? Might be clearer to someone less familiar as “local-to-global”.**

We have replaced all such instances as suggested.

- **[121] “its” should be “it’s” or “it is”.**

We are using ‘its’ as a possessive pronoun here, so this is the correct spelling.

- **[126-127] add commas or semicolons to end of each equation.**

Changed as suggested.

- **Figure 2 caption needs describing more explicitly (no mention of the points, the lines etc). What are panel (a) and (b)?**

We have added more detail here as suggested (P12 Fig. 2).

- **Could the plot colours more intuitively represent the disease? (The black line could easily be missed as the founder trees and the red/blue colours don’t automatically read as susceptible and infected landscape to me).**

We feel this is down to personal preference. In particular, the primary author is colour-blind which affects their preferred colour palette. We believe red is a fairly standard colouring for infection in epidemic models, and have worked from there for a set of contrasting colours. We note we had not mentioned the black colouring in the figure legends so have now added this (P14 Fig 4 and P17 Fig 7).

- **Some Figure captions are incomplete, Figure S1, Figure 4 and Figure 7 caption don’t describe the black line of founder trees.**

As above, we have now corrected this.

- **Figure 1 is in Materials and Methods before it’s mentioned – should be in the Results section.**

Corrected.

- **Figure 5 “Top row” in caption unneeded as figure only has one row.**

Corrected.

- **[181] “numbers infected” is unclear – maybe “number of infected”?**

Changed to ‘number of infected plants’ (P9 L181).

- **[221 – 222] Figure 2 contains blue text and a superscript which looks like it may be a draft macro – remove?**

Apologies for this! Now removed.

- **[232] “As a comparison” reads as though the examination itself is for comparison, rather than the use of the dispersal model generally.**

We have re-phrased this to make our meaning clearer (P13 L249).

- **[237] Capitalise Table (and throughout for Fig/Table etc…)**

Corrected throughout.

- **[288] “diseased” should be “diseases”?**

Corrected (P18 L318).

- **[300] oak processionary moth (no capitalisation)**

Changed (P19 L317).

**We believe this work to be of high quality and of significant importance to the field, providing novel insights into the spread of BBSC and motivating additional developments and applications. The adopted methodology is flexible enough to be applied to other economically and ecologically significant plant pests and pathogens. We thank the authors in advance for the consideration of our comments and look forward to reading the next iteration of the manuscript.**

Many thanks for these helpful comments!

**Reviewer 2**

**Best and Cunniffe present epidemiological models for disease spread in a spatial lattice, focusing on plant disease spread in fields or orchards. The topic is enormously important because of the many economically impactful diseases that affect plants throughout the world. Although many crops are certainly not arranged in a lattice, lattices do provide an excellent framework for epidemic modeling with numerous important crops, especially tree crops such as citrus. The authors nicely motivate their investigation and develop different models for the spatio-temporal epidemic process. In particular, they develop a pair approximation differential-equation model for disease spread in a lattice, and consider deterministic and stochastic predictions for a disease of citrus. They compare results with those obtained with a somewhat more traditional dispersal kernel model. I like how they incorporate founder plant infections into their lattice models.

The models, including the parameters, are clearly explained. Others have developed theoretical lattice-based models for epidemics (and see my comment below), but a highly significant aspect of the current work is that the authors took a rigorous approach to fitting their models to actual epidemic data, estimating parameters, and comparing predictions (including the heterogeneity of the predictions). They took a Bayesian approach, and utilized the ABC estimation method since the likelihood is not easily computed. This is appropriate for their complex model. Readers should be able to utilize the authors’ methodology with other models and other biological systems.**

Many thanks to the reviewer for these positive comments, we truly appreciate it! **Overall, the authors did an excellent job of summarizing the literature on modeling of spatial-lattice models, with one exception. I was surprised that the spatial-lattice epidemic modeling papers of J.A.P. Filipe and colleagues were not mentioned. I realize that there are differences with the current work, such as: Filipe primarily deals with SIS models, but the current manuscript deals with SEI models; Filipe took primarily a theoretical approach, but the current manuscript heavily deals with fitting models to data. Nevertheless, Filipe deals extensively with pair approximations, mean fields, nearest-neighbors, closure, etc. I think the authors of the current work should incorporate how they relate to Filipe’s work. Example papers include (to varying degrees of relevance): Filipe and Gibson (1998; Phil. Trans. Roy. Soc. London B); Filipe and Gibson (2001; Bull. Math. Biol.); Filipe and Maule (2003; Math. Biosciences); and Filipe and Maule (2004; J. Theor. Biol.).**

Thank you for identifying these papers – they were indeed an oversight. One in particular is very appropriate as it also fits a simplified lattice-based model to experimental data. We now cite these in the introduction (P5 L88) and make reference again in the discussion (P20 L354).

*“The closest example is that of Filipe et al. [59], who fit a purely near-neighbour lattice model to temporal experimental data of Rhizoctonia solani infection of radish plants, finding it fit their data well. Our work goes further by allowing the local-to-global parameter itself to be fitted to our dataset, to include limited spatial data in the fitting, and to take a Bayesian fitting procedure.”*

*“Only one previous study had used a lattice-based model to fit to experimental data [59], which assumed entirely near-neighbour infection, and also showed good agreement between the model and data.”* **I have just a few specific comments (referenced by line number).

143 It is fine to use an exponential dispersal kernel for this work. However, I think the authors could add some text to the Discussion about the implication of using a fat-tailed dispersal kernel. Empirical studies show about half of observed dispersal gradients have a fat tail.**

Thanks, we agree, and have added some text to the discussion (P19, L340).

*“The dispersal kernel model used here for comparison with our lattice-based model was inherited from the original study of Cunniffe et al. [61], and used an exponential dispersal kernel. The probability of transmission decays exponentially with distance, a so-called thin-tailed kernel. Fat-tailed kernels - which decay more slowly than exponentially in their tails - are often supported by empirical plant disease data (see Fabre et al. [34] for a review). Such kernels are well known to have important epidemiological consequences, most notably permitting disease wavefronts that accelerate over time [78, 79, 80]. The choice of a thin-tailed kernel in [61] was motivated by apparently highly localised transmission and a relatively constant wave speed in the experimental data, and strong support from exploratory analyses comparing alternative dispersal kernel formulations. In this context, it is therefore striking that we were able to recover a good fit to the experimental data using the local–global model, since the equivalent dispersal structure is in fact closer to a fat-tailed kernel, since the global component of dispersal corresponds to an equal probability of dispersal between any pair of trees, irrespective of distance. However, perhaps as a consequence of the limited spatial extent of the experimental plot, this did not result in substantial systematic lack of fit.*”

**203 It is not clear to me how the threshold of 0.025 was selected based on the results in Table 1. I think the authors could improve this explanation.**

This was one of the two main points that all the reviewers raised. We have now added further plots in the supplementary information showing how the posteriors change for different acceptance thresholds. These show that the posteriors seem to stabilise around the value of the chosen threshold. We also say more about this in the main text (P10 L216).

*“In the supplementary information we show posterior distributions for a number of different thresholds for comparison. We find that our chosen threshold has enough accepted fits to form `smooth' posteriors, and the distributions remain consistent for small changes to the threshold.”* **Table 2 (etc.): Bayesian usually refer to “Credible Intervals” and not “Confidence Intervals” for parameters; this better distinguishes between the frequentist interpretation of sampling distributions and the Bayesian interpretation of posterior distributions. There are two main methods for the calculation of the limits of a credible interval, which should apply to bootstraps: Equal-Tailed and Highest Posterior Density. These can differ, especially for skewed posteriors. Maybe I missed it, but I don’t think the authors mentioned the approach they took.**

This was the other main point that all the reviewers mentioned! As discussed at the top of this response, we now present the 90% smallest credible intervals (or highest density interval) for the parameter values (P11 Table 1 and P15 Table 2). **211 Supplemental figure 2 shows a NONLINEAR relation between 1/rho and beta, with close to an asymptotic relation. I think the implications of this could be mentioned.**

We have added a sentence to explicitly address this point (P11 L228).

*“We show correlations between the parameters in supplementary Fig. 2, which highlight that $\beta$ and $1/\rho$ have a strong non-linear correlation, with a much broader spread of transmission rates for high latent periods.”* **Fig. 2 legend: Not clear why there is a “Nik” superscript on “model”.**

Apologies for this (embarrassing) slip. Now removed. **353: Citation 58 gives a year of 2006 (see references). However, here the authors say 2014.**

Corrected to 2006 (P21 L390).

**Reviewer 3**

**This article develops and applies a lattice-based model using a pair approximation method to the spatial spread of a plant disease, with a case study on Bahia bark scaling in citrus. The authors successfully fit this model to experimental field data, demonstrating that it captures the disease dynamics effectively and identifies a significant component of local, near-neighbour transmission. Its performance is shown to be comparable to a more established dispersal-kernel modelling approach. From my perspective, having substantially checked the literature, I can confirm that while pair approximation models have a strong theoretical foundation in epidemiology, I could not indeed find a preceding instance where such a model was formally fitted to real-world data for a specific pathosystem. This successful application constitutes the main novelty of the work and makes it of high importance to the literature. It represents a substantial addition to the methodological toolkit of mathematical ecology and epidemiology.**

**One direct and impactful application can be that it provides a pathway for embedding realistic spatial dynamics into decision support systems. Indeed, this lattice-based pair approximation models with local:global ratio consideration and its fitting offer a deterministic framework that can realistically capture the spatial disease spread dynamics often associated with more computationally intensive stochastic models. This bridge between realism and computational efficiency is a significant step forward for optimizing disease control strategies in plant epidemiology. Therefore, I strongly recommend this paper for publication, pending revisions. My concerns are largely minor, and some are merely suggestions that can be ignored if they require undue additional work. I will specify these when it applies.**

Many thanks for these positive comments!

**Comments**

**1. (Minor) Line 100-101: “those trees initially inoculated at the start of the experiment, one row outside of the main tree stand” sounds like the founders are out of the trellis, and do not resonate well with the relationship PS+PE +PI+PF = 1. The little ambiguity should be cleared up. From my understanding, the full lattice is made of (a) a main tree stand and (b) a founder row.**

We have clarified that the founder row is outside of the main *experimental* stand of trees, which hopefully makes it clearer (P6 L119).

**2. (Minor) In line with the previous comment, although it is well described in lines 163-173, may be specifying that the dark rows stand for founders in Fig 4 and 7 captions would help “skimming readers”.**

Yes, we realise we had forgotten to include the meaning of the black cells in the figure legends and have now added these (P14 Fig 4 and P17 Fig 7).

**3. (Typo) In Eq (1), LF qI/S → LqI/S, considering lines 112-114.**

Well spotted – we have corrected this (P6 Eq. 1).

**4. (Minor) Line 122: “since each individual has 4 neighbours” assumes a Von Neumann neighbourhood. To what extent would the results change if a Moore neighbourhood (8 neighbours) was used? This questions the fundamental distance considered for neighbouring, specifically whether a unit of distance is distinct from that same unit multiplied by √ 2 (Pythagoras). Have you tested both configurations to see how they impact the model approximation and calibration? If further exploration is not deserved, this limitation deserves at least a brief discussion in the methods.**

This is a good point, and links to the passage in the discussion where we talk about using multiple interaction scales. We have expanded that section to talk about this more (P19 L337).

*“Here we used a simple von Neumann neighbourhood of four near-neighbours on a square lattice, but alternative neighbourhood definitions could also be used to alter the scale of local infection.”*

**5. (Typo) Eq (6) γ → ρ.**

Another good spot – corrected (P7 Eq. 6).

**6. (Major) The introduction of the local:global infection parameter control L looks quite simplistic as it is curious that it is a simple ratio. Can it be more elaborated upon its introduction? More explicitely, the distance structure is ignored and all “global” infections seem to have the same probability; all non-local infections are lumped together, ignoring spatial decay. I suggest the single L to be introduced in the singlet model as a two-bin approximation neighbours vs “everyone-else”, which is acknowledgely useful for minimal models but might miss clustering effects and realistic spatial spread. Then maybe it can become a better approximation through the pair closure and/or the data fitting.**

This is a point we had acknowledged, in particular with the second paragraph of the discussion entirely devoted to considering the impact of this simplicity, and was also mentioned directly at a few relevant points in the results. We have added a sentence when the model is first discussed to stress the point further (P6 L130).

*“We stress here that ‘local’ infections are purely near-neighbour, and ‘global’ infections are random/mean-field, such that L = 0.5 means that half of an individual’s infectious contacts are from neighbouring sites and half from anywhere else on the lattice.”*

**7. (Minor) Lines 189-191: I do not understand “For these purposes (...) tree stand”.**

This is just to make the point that since the experimental data does not include non-founder trees, we need to adjust the output of the model to not include the row of founder trees. We have tried to re-phrase this to make it clearer (P9 L199).

**8. (Minor) Line 199: The link is truncated.**

Corrected (P10 L212).

**9. (Minor) Line 203: Which threshold? The sentence “Table 1 shows how the number of successful fits depends on this threshold” cannot stand alone. Additionally, although the threshold was introduced at lines 191-192, it needs more details; otherwise, it is difficult to understand.**

This was one of the two main points that all the reviewers raised. We have now added further plots in the supplementary information showing how the posteriors change for different acceptance thresholds. These show that the posteriors seem to stabilise around the value of the chosen threshold. We also say more about this in the main text (P10 L217).

*“In the supplementary information we show posterior distributions for a number of different thresholds for comparison. We find that our chosen threshold has enough accepted fits to form `smooth' posteriors, and the distributions remain consistent for small changes to the threshold.”*

**10. (Typo) Fig. 1 (c) subcaption: → Latent period, 1/ρ**

Corrected.

**11. (Typo) Fig. 2: Looks like the author’s comment (Nik) is still visible in the caption.**

Yes, apologies for this. Corrected.

**(Minor): If the authors deem it useful, I would suggest adding a legend to Fig. 2 (a) as well as a posterior predictive mean (PPM) line. A mean curve is even more relevant for Fig. 3 (a) and Fig. 6 left, because of the variation. Fig. 2 caption can also be made richer for “skimming readers”.**

We have chosen not to include the suggested PPM line – we feel it is more in the spirit of the Bayesian approach to show the variation in outputs from the 100 randomly selected posteriors. We have, however, expanded the caption as suggested.

**12. (Major) Fig 4.: The choice of L, β, βF , ρ, δ out of their 95% confidence interval (Table 2) for the simulation is curious. What justifies this choice?**

This is in part connected to us reporting the confidence intervals instead of the credible intervals. With the newly generated posterior distributions we have now altered these anyhow and have taken values from within the 95% credible intervals for each parameter.

**13. (Minor) Lines 235-236: The notion of threshold here is still unclear, as previously.**

As above, hopefully this is clearer now with the additional content.

**14. (Minor) Line 239: What does “only 5 parameter sets accepted where α > 5” mean?**

We have re-phrased this to be clearer, although we note that the actual number has changed now that we have expanded the overall number of model runs (P13 L256).

**15. (Major) Lines 245-248: We need a better explanation of why the PPC of the dispersal model look visually more similar to the stochastic simulation of the lattice model than the actual dispersal model. Additionally, I suggest a single, robust measure to compare the ability of the lattice and dispersal models to fit the data: the Earth Mover’s Distance (EMD) applied to monthly snapshots. The EMD quantifies the minimum cost to transform one spatial distribution into another, providing a powerful metric for overall spatial dissimilarity[Rubner et al. 2000: The earth mover’s distance as a metric for image retrieval. IJCV 40(2)]. Since it accounts for the full spatial structure, it offers a more comprehensive comparison than metrics based solely on global or row-wise infection proportions (Figs. 3 and 6). Hence, plotting EMD(lattice, data)(t) and EMD(dispersal, data)(t), t in months, on the same graph would allow for a direct visual and quantitative discussion of their relative performance over time. While this is not a mandatory request, I believe its inclusion would significantly strengthen the manuscript’s results and provide a concrete basis for discussing the advantages of the lattice model. The EMD could, of course, be substituted with a similar spatial metric, such as a class-wise Hausdorff distance or normalized Hamming distance [Hamming 1950: Error detecting and error correcting codes. The Bell System Technical Journal 29(2)]. Given the nature of the experimental data, latently infected trees can be considered in either the S or I class while assessing the EMD, Hausdorff, Hamming, or a similar metric. This necessary misclassification could influence the results and may be worth a brief mention.**

This is a very interesting idea, and we did give it some thought, but we felt it was really beyond the scope of what we are wanting to achieve in this paper. These plots about the spatial spread of the infection were really there to be a visual guide, and helped highlight what is probably the main pitfall of the lattice model – that in a large population it will over-predict long-range infections and under-predict mid-range ones. We now include the EMD technique as a possible alternative approach to including more spatial information in the ABC fitting statistic over and above the approach by Minter that we already referenced in the discussion (P20 L365).

*“…or the Earth Mover’s Distance [92] between the simulation runs and the spatial data”.*
